# In Vitro Assessment of the Prebiotic Potential of Xylooligosaccharides from Barley Straw

**DOI:** 10.3390/foods12010083

**Published:** 2022-12-23

**Authors:** Cristina Álvarez, Alberto González, Ignacio Ballesteros, Beatriz Gullón, María José Negro

**Affiliations:** 1 Advanced Biofuels and Bioproducts Unit, Department of Energy, Research Centre for Energy, Environment and Technology (CIEMAT), 28040 Madrid, Spain; 2 Department of Chemical Engineering, Faculty of Science, University of Vigo (Campus Ourense), As Lagoas, 32004 Ourense, Spain

**Keywords:** prebiotic activity, in vitro digestion, barley straw, xylooligosaccharides

## Abstract

Barley straw was subjected to hydrothermal pretreatment (steam explosion) processing to evaluate its potential as a raw material to produce xylooligosaccharides (XOS) suitable for use as a prebiotic. The steam explosion pretreatment generated a liquid fraction containing solubilised hemicellulose. This fraction was purified using gel permeation chromatography to obtain a fraction rich in XOS DP2-DP6. The sample was characterised through analytical techniques such as HPAEC-PAD, FTIR and MALDI-TOF-MS. The prebiotic activity was evaluated using in vitro fermentation in human faecal cultures through the quantification of short-chain fatty acid (SCFA) and lactate production, the evolution of the pH and the consumption of carbon sources. The total SCFA production at the end of fermentation (30 h) was 90.1 mM. Positive significant differences between the amount of XOS from barley straw and fructooligosaccharides after incubation were observed.

## 1. Introduction

In recent decades, agroindustry has begun to orientate itself towards a sustainable transformation focused on the recovery of by-products to produce high-value-added products, such as sustainable ingredients for food and beverages, animal feed and pharmaceuticals. The valorisation of these waste and by-products has led to more sustainable and environmentally friendly production, introducing the concept “circular economy”. However, the circular economy is more than recycling waste; it means transforming what was considered waste into high-value resources. Lignocellulosic biomass (LCB) is the most abundant carbon-based renewable resource. Its worldwide production is estimated at 1.3 × 10^10^ metric tons per annum [1]. LCB has a valuable fraction containing hemicellulose, which can be transformed into xylooligosaccharides (XOS) composed of xylose units. Xylooligosaccharides are oligomers made up of a D-xylose backbone-joined by β-(1→4)-linkages that can include branched structures with substituents such as arabinose, glucuronic acid, an acetyl group or phenolic at C2 or C3 positions on the xylose units [2]. Many lignocellulosic residues have been studied to obtain XOS, such as barley straw, wheat straw, sugarcane bagasse, rice husk, almond shell, etc. [3,4,5,6,7,8]. In recent years, XOS with a low degree of polymerization (DP) have received significant attention as prebiotic compounds. *Prebiotics* are defined as “a substrate that is selectively utilised by host microorganisms conferring a health benefit” [9]. The main end-products of prebiotic degradation are SCFAs like acetate, propionate and butyrate, which perform different functions that have positive effects on health [10]. Prebiotics play a significant role in preventing diseases in cardiovascular systems, gastrointestinal tracks and endocrine systems, as well as cancers. XOS greatly boost lipid metabolism, offer anti-inflammatory effects, increase effective calcium absorption and protect against cardiovascular diseases [9]. XOS have biological properties such as antioxidant, antibacterial and immunomodulation actions that help in reducing diabetes and colon cancer risks [2,11,12,13,14].

In the last few years, consumers have increased their demand for these types of compounds, creating a growing market for foods containing prebiotics. In particular, the XOS global market was estimated at 61 million USD in 2019 and is expected to grow by 4.1% annually to reach a value of 81 million USD in 2026 [15]. In Europe, the organisation responsible for authorising food ingredients is the European Food Safety Authority (EFSA). In 2018, EFSA Panel on Dietetic Products reported that enzymatically-produced XOS from corncobs (novel food) were safe for use by the general population and established dosage levels [16]. To access its carbohydrates, LCB must be pretreated to alter its structure and divide it into the main components, which can be separated. The type of pretreatment of hemicellulose depends on its intended end use.

Among the physicochemical treatment methods, steam explosion (SE), carried out at high pressure and temperature, typically 180–240 °C, has proven a successful option. SE is a hydrothermal pretreatment that combines mechanical and chemical effects which lead to better accessibility and digestibility of cellulose and further hydrolysing hemicellulose into oligosaccharides, monosaccharides and products of sugar degradation such as furfural and 5-hydroxymethylfurfural (HMF) are generated [17,18]. Moreover, these undesired products include non-saccharide fractions derived from extractives, acid-soluble lignin and inorganic components are present.

Hence, the purification of XOS to remove monosaccharides and other undesired compounds is an indispensable step in creating XOS products sufficiently pure for use in foods. Because high-purity products have a more powerful impact on the biological function [19], a downstream step is required. Several purification techniques such as solvent extraction, adsorption techniques, membrane separation and chromatography have been evaluated [2,14]. In this study, gel permeation chromatography has been used as it is commonly employed and easily adapted. This work also proposed isolating the XOS (DP2-DP6) generated in the steam explosion pretreatment of barley straw (BS), which accounts for 40% of the oligosaccharides with DP2-DP6 purified using gel permeation chromatography. This methodology has the advantage of not needing an enzyme hydrolysis step, only a purification step. These XOS were characterised using HPAEC-PAD, FTIR and MALDI-TOF-MS. Finally, an in vitro assay was done to check the prebiotic activity of this sample. This approach could be a suitable strategy to increase the value of this unexploited waste biomass.

## 2. Materials and Methods

### 2.1. Raw Material and Pretreatment

The material used in this study was an agricultural waste, barley straw (*Hordeum vulgare*), supplied by the Renewable Energy Development Centre (CEDER-CIEMAT) (Lubia, Soria, Spain). The biomass composition analysis was repeated three times in accordance with the methodology described by Sluiter et al. [20]. The results showed the following average composition (in % dry weight basis): 39.0% cellulose, 27.3% hemicellulose (22.1% xylan, 1.3% galactan 3.6% arabinan and 0.3% mannan), 1.7% acetyl groups, 18.9% total lignin, 13.4% extractives and 3.9% whole ash.

The barley straw (BS) was subjected to SE pretreatment to produce XOS. The SE pretreatment was carried out using a prototype unit located at CIEMAT. This SE unit operates in batch mode and consists of a steam generator, a 10 L capacity reactor and a discharging cyclone [21]. The generator supplies saturated steam to the interior of the reactor, where the biomass is subjected to controlled temperature, pressure and time conditions. Once the desired temperature has been reached and the set residence time has elapsed, the unit is depressurised rapidly by opening its lower valve and passing the pretreated material to the discharge cyclone [22]. The operating conditions (180 °C and 30 min) were chosen as previous studies indicate they result in the maximum recovery of XOS in the liquid fraction [23].

After pretreatment, the material (slurry) was recovered, cooled and filtered to separate the water-insoluble solids and the liquid fraction. The liquid fraction (prehydrolyzate) contained the solubilised hemicellulose fraction. This liquid fraction was further subjected to a cleaning step with ion exchange resin (Microionex MB200 (Rohm-Haas Copenhagen, Denmark), following the method previously described by Negro et al. [24] To Remove Any Degradation Compounds Of Sugar And Derivate Lignin.

### 2.2. Fractionation and Purification of the Liquid Fraction

After the cleaning step, the XOS (DP2-DP6) present in the liquid fraction obtained from the pretreatment of BS were separated. The separation process was carried out using gel permeation chromatography. The separation process used column packing Bio-GEL P2 (Bio-Rad, Hercules, CA, USA), which is made of a synthetic polyacrylamide polymer capable of separating molecules with a molecular weight between 100 and 1800 Da, using 0.1 M acetic acid as a mobile phase with a flow rate of 0.1 mL/min and at room temperature. The column is connected to an automatic injector (Waters 717 plus autosampler) together with a pump (Waters 600 controller) and a refractive index detector (Waters 2414) (Waters Corporation, Boston, MA, USA).

The purification process was carried out by collecting three fractions with different molecular weights: the first one containing the compounds with a high degree of polymerization, the second fraction containing our target compounds (DP2-DP6) and the last fraction containing the monosaccharides and other compounds with a low DP present in the sample. This step was repeated several times to obtain enough to do the assays.

In order to optimise collection times, the samples were freeze-dried and regenerated in water before being analysed by HPLC-IR and HPAEC-PAD to check if the sample contained our compounds of interest.

**Figure 1:**
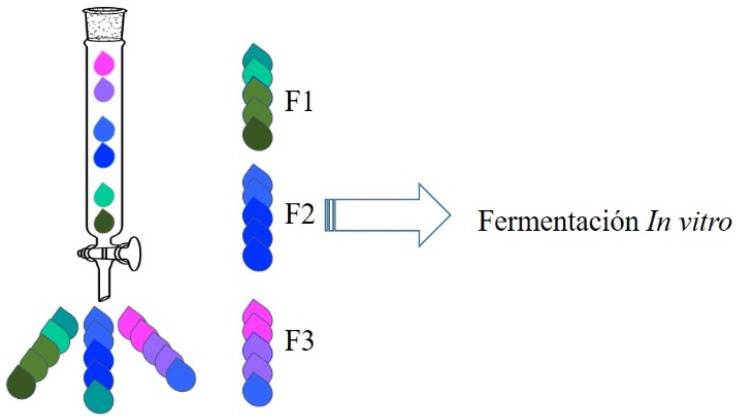
Scheme of fractionation of prehydrolysate fraction.

In the purification step, three fractions were obtained according to their molecular size, following the scheme shown in Figure 1. The first fraction (F1) contained the compounds with a degree of polymerization higher than 6 and was collected from minutes 140 to 230. This fraction can be subjected to enzymatic hydrolysis to obtain XOS with a low degree of polymerization, thus increasing the amount of XOS in the process [25]. The second fraction (F2) contained compounds with a degree of polymerization of DP2-DP6 and was collected from minutes 230 to 315. Finally, the third fraction (F3), was where monosaccharides were collected. In addition, low molecular weight compounds, such as formic and acetic acids, etc., present in the sample were collected in this fraction. This fraction was collected from minutes 315 to 350.

### 2.3. Fermentation of XOS

The F2 sample of XOS was fermented using a final concentration of 8 g oligosaccharides/L and 10 g/L of fructooligosaccharides (FOS) as a positive control. The faecal inocula were donated from three healthy volunteers (25–40 years old) who had not taken antibiotics for at least three months preceding the study. The inocula were diluted in reduced physiological salt solution in a ratio of 10 *w*/*v*. All additions and inoculations were carried out in an anaerobic cabinet (5% H_2_, 10% CO_2_ and 85% N_2_).

Experimental conditions such as the preparation of the nutrient base medium, faecal inocula and anaerobic conditions were prepared following the method described by Gullón et al. [26]. The fermentation assays were carried out at 37 °C for 30 h without shaking. Samples were measured at 0, 4, 7, 10, 24 and 30 h from the fermentation broths, and centrifuged at 5000× *g* for 5 min. High-performance liquid chromatography (HPLC) was used to determine the SCFAs, lactate production and oligosaccharides consumption. Moreover, the pH of the medium was measured.

### 2.4. Analytical Methods

#### 2.4.1. Sugars, Phenolic Compounds and Degradation Products

The HPLC used for measuring sugars in monomeric form (hexoses and pentoses) was performed using a Waters 2695 liquid chromatograph with a 2414 refractive index detector (Waters Corporation, Milford, MA, USA) and a CarboSep CHO-782 Lead column (Transgenomic, Omaha, NE, USA) operating at 70 °C with Mili-Q water as the mobile phase with a flow of 0.5 mL/min. The total oligosaccharides in the liquid fraction were determined by the difference between the concentrations of total sugars and monosaccharides in the liquid fraction following mild acid hydrolysis with H_2_SO_4_ 4% (*w*/*w*) for 30 min at 121 °C.

The phenolic compounds, together with degradation products such as 5-hydroxymethylfurfural and furfural, generated during the SE pretreatment were determined by HPLC using an Agilent 1100 series chromatograph (Agilent Technologies, Santa Clara, CA, USA) equipped with an Agilent 1200 series Diode-Array and a Transgenomic ICSep ICE-COREgel 87H3 column combined with an analytical guard column (Transgenomic, Omaha, NE, USA) at 75 °C. The mobile phase was 89% 0.005M H_2_SO_4_ and 11% acetonitrile (0.7 mL/min).

#### 2.4.2. Determination of Short Chain Fatty Acids (SCFAs)

The determination of SCFAs obtained in the in vitro fermentation of the prebiotic xylooligosaccharides was performed using an Agilent 1200 series chromatograph coupled to a refractive index detector (Agilent Technologies, Santa Clara, CA, USA). For this purpose, an Aminex HPX-87H column (Bio-Rad, Hercules, CA, USA) was employed at 50 °C using a mobile phase of 0.003 M sulfuric acid in isocratic mode at a flow rate of 0.6 mL/min.

#### 2.4.3. Structural Characterisation of Xylooligosaccharides

##### High-Performance Size Exclusion Chromatography (HPSEC) and High-Performance Anion Exchange Chromatography with Pulsed Amperometric Detection (HPAEC-PAD)

HPSEC was carried out using a Waters chromatograph (Milford, MA, USA) equipped with a 2414 refractive index detector to analyse the xylooligosaccharides quantitatively. Three gel-filtration columns in series, Shodex KS-802 (10,000 Da), Ultrahydrogel 120 (5000 Da) and TSK-gel (G-Oligo-PW) (3000 Da), were used.

HPAEC-PAD chromatograph of Dionex System ICS2500 Dionex System (Dionex Corporation, Sunnyvale, CA, USA) with a pulsed amperometric detector (ED50 electrochemical detector) with a gold electrode was used for quantitative analysis. Both techniques followed the methods described by Álvarez et al. [4]. The calibration was done with xylooligosaccharides (DP2-DP6) from Megazyme International (Bray, Ireland).

##### Matrix-Assisted Laser Desorption/Ionization-Time of Flight-Mass Spectrometry (MALDI-TOF-MS)

The F2 sample was analysed with MALDI-TOF using an Ultraflex workstation (Bruker Daltonics, Bremen, Germany) equipped with a nitrogen laser (λ = 337 nm). Measurement was performed in positive refractor mode for a mass range of 500–2200 *m*/*z*, following the methodology described by Gullón et al. [5].

##### Attenuated Total Reflection-Fourier Transformed Infrared Radiation (FTIR) Spectroscopy

FTIR spectra were measured in a Spectrum Two FT-IR model Perkin Elmer Spectrometer (Perkin Elmer, Boston, MA, USA). Spectra were collected in the 4000–400 cm^−1^ spectral range at a resolution of 4 cm^−1^ with the scans repeated 8 times. This technique was used for the characterisation of the F2 sample.

### 2.5. Statistical Analysis

Statistical analysis was carried out using Statgraphic Centurion XVII.I-X64 for Windows. Analysis of variance (ANOVA) and a *post hoc* Least Significant Difference (LSD) test of the multifactorial analysis was used to determine the significant differences between experiments. The level of significance was set at 95%.

## 3. Results and Discussion

### 3.1. Liquid Fraction from Steam-Exploded Barley Straw

The solubilised hemicellulose was recovered in the prehydrolyzate after pretreatment with a steam explosion (180 °C–30 min). This solubilised hemicellulose was mainly in an oligomeric form. In the case of xylose-derived sugars, the concentration of xylooligosaccharides accounted for 23.1 g/L, whereas the concentration of xylose was only 3.1 g/L. Glucose present in the liquid fraction was measured in monomeric form (0.8 g/L) and oligomeric form (5.2 g/L). The concentration of the other sugars, such as arabinose, mannose and galactose, were 2.1, 0.07 and 0.6 g/L, respectively. The oligomeric form of these sugars was 1.2, 0.4 and 1.6 g/L, respectively. Approximately 7.6 g of XOS (DP2-DP6) per 100 g of the raw material had been generated using steam explosion pretreatment of BS at 180 °C–30 min. These compounds made up 40% of the carbohydrates present in the sample. This value is higher than that of poplar (66.4 g of XOS in 1 kg of raw material) produced by an integrated process involving hydrothermal and hydrotropic acid pretreatment [27].

In addition to sugars, degradation products were also found in the phehydrolyzate. Due to pentose and hexose degradation, furfural (1.0 g/L) and HMF (0.1 g/L) were detected. Formic acid (0.8 g/L) results from furan degradation and acetic acid (1.7 g/L) results from releasing acetyl groups of hemicellulose. The lignin degradation products *p*-coumaric acid, vanillin and ferulic acid were also detected at low concentrations of 30, 40 and 34 mg/L, respectively.

### 3.2. Fractionation Step

As described in the previous section, fractionation was carried out by gel permeate chromatography. Before fractionation, the cleaning process was performed. The cleaning process did not affect monosaccharides; however, the concentration of phenolic compounds and carbohydrate degradation products decreased markedly. For example, furfural and HMF decreased from 1.0 and 0.10 g/L to 0.15 and 0.02 g/L, respectively.

**Figure 2:**
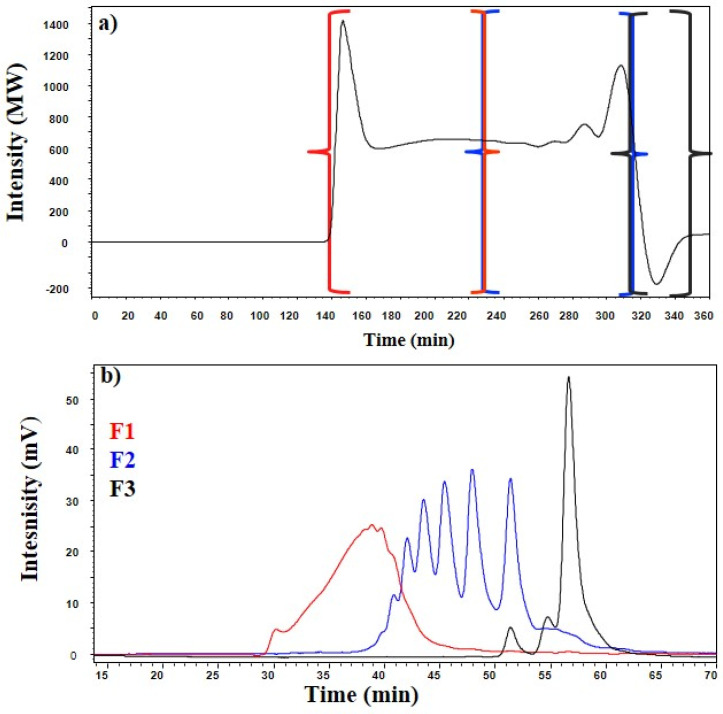
Profile of elution of prehydrolyzate from steam-exploded barley straw (**a**) chromatogram obtained by HPSEC Bio-Gel P2, (**b**) distribution of XOS analysed in. F1, F2 and F3 fractions.

The fractionation process was optimised using the gel permeate chromatography technique until a good separation of the DP2-DP6 compounds was achieved. In order to meet this objective, three fractions of the pretreated BS were collected (Figure 2).

These three fractions were collected according to their molecular size: the first fraction (F1) containing the highest molecular weight compounds > DP6, a second fraction (F2) containing the compounds with a DP of DP2-DP6. Finally, F3 was collected, which included the monosaccharides and other molecules with a low DP produced during pretreatment.

The three separated fractions (after freeze-drying and regeneration in ultra-pure water) were analysed by size exclusion chromatography, establishing the molecular weight range described in the previous section. Figure 2b shows the molecular weight distribution of oligosaccharides (major compounds) of DP6, compounds of DP2-DP6 and low polymerisation compounds (or monosaccharides) contained in each of the three fractions analysed by HPSEC.

Fraction F1 contains the compounds whose molecular weights are >1000 Da. Fraction F2 includes compounds in the range of 300 to 1000 Da and fraction F3 contains compounds with molecular weights below 300 Da, monosaccharides making up the majority of the compounds in this fraction.

In addition, the sugar content in each fraction was determined quantitatively by total hydrolysis with sulfuric acid. Table 1 shows the composition of the carbohydrates present in each fraction obtained by separating fraction liquid using the Bio-Gel P2 column.

The highest glucose concentration is found in the F1 fraction, either in the form of gluco-oligosaccharides or linked to some high molecular weight xylooligosaccharides. Xylose derivatives are found mainly in the F2 fraction. Since this separation aimed to obtain the greatest possible amount of xylooligosaccharides in a unique fraction, this goal was achieved. As noted, the third fraction (F3) was mostly monosaccharides.

**Figure 3:**
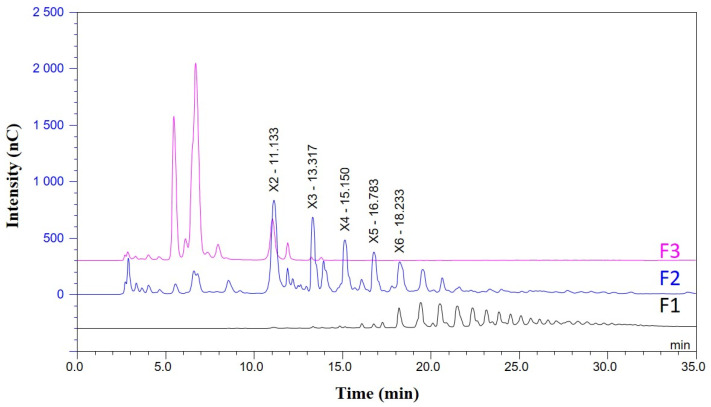
Distribution of XOS analysed by HPAEC-PAD in each fraction obtained by gel permeation chromatography.

Figure 3 shows the chromatograms of each of the fractions. Fraction F1 contained compounds with a degree of polymerisation of DP6-DP15. F2 showed the following composition of xylooligosaccharides: 2.3 g/L xylobiose (X_2_); 2.1 g/L xylotriose (X_3_); 2.4 g/L xylotetraose (X_4_); 2.3 g/L xylopentose (X_5_) and 2.0 g/L xylohexose (X_6_). These results can also be expressed as 6.6 g of XOS with DP2-DP6 (expressed as xylose) per 100 g of raw material. The last fraction included (F3) corresponds to the elution of the monosaccharides and a certain amount of xylobiose (0.5 g/L). Obtaining XOS is directly related to the prehydrolizated pretreatment, it is not necessary to use enzymes to produce XOS with a low degree of polymerisation. This strategy provides an economic advantage. XOS from steam explosion pretreatment could be incorporated into an economically viable integrated biorefining process [28].

The purification of a liquid fraction by size exclusion chromatography separated a fraction (sample F2) with a degree of purity in XOS of 81%. This degree of purity is sufficient to be used as a commercial prebiotic (75–95%) [29]. The purity shown in this work is similar to that obtained by Cara et al. [28], who published purities between 82 and 90% by applying this methodology to the liquid fraction obtained from pruning residue pretreated by hydrothermal pretreatment. These authors managed to separate oligosaccharides with DP7–25.

In prior studies, this purification method has efficiently separated and purified the xylooligosaccharides produced by biomass autohydrolysis. Xiao et al. [30] used this method to separate XOS from pretreated bamboo, obtaining separated samples of X_2_, as well as acetylated xylooligosaccharides between X_3_ and X_6,_ which showed outstanding purities of 94.5%, 93.2%, 90.8%, 88.4% and 85.2%, respectively, with an acceptable recovery (71–86%). Moniz et al. [31] also used this method to obtain XOS from corn straw, fractionating it into three main product groups: those containing small polysaccharides and high oligosaccharides (DP > 39); those containing medium oligosaccharides with polymerisation of DP3-DP23, the target being substituted XOS (DP 4–21); and products with disaccharides, monosaccharides and bioproducts (such as furans and acetic acid). The fractions which contained DP4–DP21 made up about 35% of the total mass recovered, with purity for all fractions ranging between 77 and 85%. This division allowed us to obtain samples for different applications, such as, for instance, polysaccharides to be used to produce hydrogels and activated carbons [32].

### 3.3. Structural Characterisation of Xylooligosaccharides in F2

The effect of xylooligosaccharides derived from hemicellulose depends on its structure (degree of polymerisation, type of bonding, monomers) [33]. Knowledge of the structural characteristics of the material is essential since the biological properties depend on the distribution of their molecular weight and their degree of substitution.

#### 3.3.1. MALDI-TOF-MS

The MALDI-TOF-MS technique was used to learn more details about both the chain length and the nature of the branched compounds contained in the xylooligosaccharides. Table 2 shows the structural attributes of the *m*/*z* signals in the MALDI-TOF-MS spectra (included in supplementary material, Figure S1) for the xylooligosaccharides.

The molecular ions of oligomers were identified as potassium and sodium adducts. This technique confirms the presence of xylooligosaccharides with a low degree of polymerisation. The spectrum (Figure S1 of the supplementary material) shows how the fractionation of the sample was performed, finding compounds with a degree of polymerisation of DP2-DP6, which was the objective. However, the presence of linear XOS of up to DP8 was also detected. In the mass spectrum, signals attributed to a series of compounds consisting of acetylated XOS with one acetyl group between DP2 and DP7 and two acetyl groups with DP4-DP6 were also detected. The spectrum acquisition is recorded from the *m*/*z* ratio greater than 400, so this technique does not confirm the presence of xylobiose, previously detected by HPLC. MALDI-TOF-MS can show how the acetyl groups are attached to the xylooligosaccharides.

This technique can determine how well the purification process is done. The literature contains examples of the liquid fraction from the steam explosion of BS containing varying degrees of polymerization ranging from monosaccharides to oligosaccharides with DP15 [34].

Knowing the structural characterisation is crucial since substitutions in oligosaccharides can affect their behaviour during fermentation and thus modify the prebiotic potential [35]. The literature describes how branched oligosaccharides with acetyl groups performed better in producing beneficial bifidobacteria than non-substituted ones [36].

#### 3.3.2. FTIR

**Figure 4:**
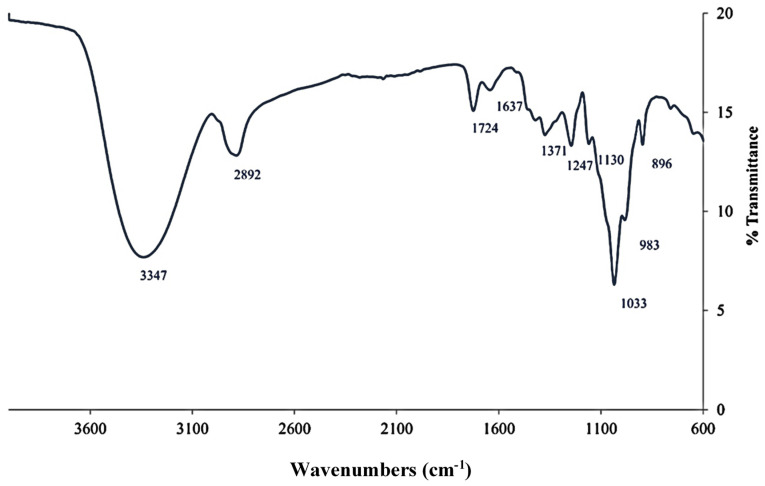
Spectrum FTIR.

FTIR spectroscopy was used to determine the functional groups present in the sample and their structure, as well as elucidate complex and intermolecular interactions. The assignment of transmission spectrum bands was carried out according to the literature [37,38,39,40,41,42,43] since this spectrum shows the typical transmittance bands for hemicellulosic oligosaccharides. Its spectra are plotted in Figure 4.

Signals around 1724, 1371 (C-H bonds in hemicelluloses) and 1247 cm^−1^ were attributed to the presence of acetyl groups. The 1724 cm^−1^ band is related to the stress vibration due to the carbonyl bonds of the acetyl groups of xylans in hemicelluloses [37] or carboxyl groups of ester bonds in cinnamic acids such as ferulic acid (typical of herbaceous plants). The band around 1430 cm^−1^ corresponds to C-O stretching and bending of C-H and/or O-H in the hemicelluloses [38]. The signals around 1247 cm^−1^ indicate the vibrational band of the single bond C-O stretching band related to the acetyl groups present in the hemicelluloses [37,39]. The presence of acetyl groups confirms the results obtained by the MALDI-TOF-MS technique. The low-intensity shoulder, which appears at 1130 cm^−1^, is used to identify oligosaccharides containing arabinoses, as it is characteristic of these chains [40,41]. The region between 1130 and 920 cm^−1^ corresponds to the xylans. The maximum band observed at 1033 cm^−1^ is attributable to the stretching of C-C, C-O or C-OH bending of the glycosidic linkage in hemicellulose (COC) [42], indicating the predominance of xylan oligosaccharides [43].

Two weak tails identify the presence of the arabinoxylan side chains at 1130 and 983 cm^−1^. In particular, the band at 983 cm^−1^ is attributable to the vibrations of the arabinoxylan side chains, being sensitive to the location of the substituent [42]. This finding confirms that the sample contains arabinoxylans, as shown in Table 1. In the region of 950–700 cm^−1^, a small sharp band at 896 cm^−1^ indicates the configuration of the (1→4) glycosidic bonds between the xylopyranose units in the xylan chains [42].

### 3.4. Analysis of Prebiotic Properties

This section shows the results from analysing the prebiotic properties of XOS obtained from BS pretreated by steam explosion. The previously freeze-died sample was used as a carbon source by using intestinal microbiota.

A prebiotic evaluation was performed by examining the formation of SCFAs and the consumption of the substrate. The results are compared with a sample of FOS used as a positive control since there is scientific evidence of its prebiotic character.

During fermentation, the pH in the sample dropped from an initial 7.4 to 5.9 (Table 3). Two stages can be distinguished in the lowering of the pH during fermentation, one where this decrease is significant (first 10 h) due to the growth and/or metabolic activity of intestinal bacteria, and a second where the pH remains constant until the end of fermentation. The pH drops were 1.5, demonstrating that faecal microbiota readily utilised the substrate.

In the fermentation medium, a mixture of short-chain fatty acids was generated, mainly acetic, butyric and propionic acids, which may have beneficial properties in the organism. The quantitative changes in the SCFAs observed in the in vitro cultures are summarised in Table 3. In this sample, 90.1 mM of SCFAs were obtained at 30 h of fermentation. This value is similar to that described in Álvarez, [34] which also used barley straw.

Acetic acid accounted for 72.1% and 56% of the total SCFAs for F2 and FOS, respectively. The highest concentrations of this acid were obtained at 30 h (65.2 mM) in fermentations, showing significant differences (*p* ≤ 0.05) as compared to FOS at the same fermentation time (38.3 mM). The results observed in this work are lower than in a previous study using XOS derived from corn stover, vine shoot, or *Eucalyptus nintes* wood at similar times [40,44,45], but higher than those obtained from corn straw [46]. Acetic acid plays a key role in the regulation of epithelial cell division. Some studies also link it to different intestinal functions such as increased colonic blood flow [47]. This acid represented approximately 70% of the total SCFA. This amount is similar to that observed in the fermentation of oligosaccharides from olive tree pruning [48]. This acid is the primary source of energy for the colony of epithelial cells and its increased production by gut bacteria has been linked to a reduced incidence of colon cancer [49]. The production of this acid was not observed until 7 h of fermentation. Finally, it reached a concentration of 12.5 mM after 24 h of incubation. While, during the same period, Dávila et al. [40] observed more of this acid when evaluating the prebiotic effect of refined XOS from vine shoot, the amount in this study is higher than that obtained by Buruiana et al. [44].

Another SCFA that is very important in the gut environment is propionic acid. In this case, 12.2 mM were produced at 30 h. In many assays described in the literature, there is a controversy as to whether propionic acid was the second or third main organic acid produced in fermentation [34,40,44,45]. These differences might be related to the source and chemical structure of XOS. Yet another key factor is the enormous complexity and inter-individual variability in the human gut microbiota, making the fermentation profile different for each person. Currently, establishing microbial modulation strategies presents major challenges [50].

Lactic acid was also produced during fermentation. This acid was generated mainly during the first 10 h of fermentation. After that time, the concentration decreased and even disappeared completely in healthy subjects. This happens because some bacteria can consume it to generate other metabolites such as acetate or propionate [51].

From the data shown in Table 3, it can be seen that the ratio between acetic and propionic acids (A/P) in F2 (5.2) is higher than that achieved by FOS (3.0) at 24 h of fermentation. This ratio is associated with lipid reduction in the liver and/or cholesterol synthesis [52].

Finally, another parameter that was monitored was XOS consumption during fermentation. Fermentation rate and SCFA profiles vary depending on the DP, substituents with linear and arabinose substituted XOS being the fastest compared to acetylated ones [35]. With regard to the assimilation of XOS during the first 10 h of incubation, 95% of the initial oligosaccharides were consumed according to the concentration profiles determined for SCFA. This substrate is suitable as a carbon source in fermentation and the rapid consumption was due to the elimination of branched side chains of XOS. The chain length of XOS is an important parameter in the determination of that consumption. The literature showed studies that suggest that oligosaccharides with branched and β (1–4) glycosidic linkages are slow to degrade [53]. This agrees with the results obtained by Álvarez et al. [34] where branched XOS from BS in samples with up to DP9 and DP15, 70% of the initial amount was consumed at 10h. Other important variables in the fermentation model are the composition of the initial microbiota and experimental conditions tested [44].

## 4. Conclusions

Lignocellulosic materials are an excellent source of hemicelluloses susceptible to extraction by pretreatment such as steam explosion (autohydrolysis). In the study, this pretreatment produced a liquid fraction, which contained XOS, degradation products and phenolic compounds. XOS must be very pure to be used commercially, so a purification step was performed using gel permeation chromatography (Bio-GEL P2). Finally, 6.6 g of 81% pure XOS (DP2-D6) was obtained in the process.

Different techniques such as MALDI-TOF-MS and FTIR were needed to specify the structural features of the solubilised products derived from hemicelluloses. These techniques confirmed the presence of oligosaccharides made up of unsubstituted pentose oligomers in the range of DP4-DP8. Mono and di-acetyl compounds were detected.

The results of this work indicate that XOS from pretreated BS can generate interesting prebiotic substrates which produce SCFAs as a result of the fermentation of prebiotics by gut microbiota. These products can have multiple potential applications as ingredients for nutraceuticals.

## Figures and Tables

**Table 1:** Composition in oligomeric and monosaccharide form (g/L) of fractions collected by gel permeation chromatography.

Fraction	Oligosaccharides (g/L)			Monosaccharides (g/L)		
	GOS	XOS	AOS	Glucose	Xylose	Arabinose
F1	4.8	8.8	1.0	0	0	0
F2	0.1	10.9	0.4	0.8	0.4	0.2
F3	0	0.16	0.2	0.3	3.0	2.0

**Table 2:** Structural assignment of the mass signals obtained by MALDI-TOF-MS.

*m*/*z*	Structure	*m*/*z*	Structure
569	[P_4_Na]^+^	833	[P_6_Na]^+^
611	[P_4_AcNa]^+^	875	[P_6_AcNa]^+^
653	[P_4_Ac_2_Na]^+^	917	[P_6_Ac_2_Na]^+^
701	[P_5_Na]^+^	965	[P_7_Na]^+^
743	[P_5_AcNa]^+^	1007	[P_7_AcNa]^+^
785	[P_5_Ac_2_Na]^+^	1097	[P_8_Na]^+^

**Table 3:** Mean values of lactic acid, SCFA concentrations (mM) and pH during fermentation media.

Carbon Source	Time (h)	Lactic Acid (mM)	Acetic Acid (mM)	Formic Acid (mM)	Butyric Acid (mM)	Propionic Acid (mM)	SCFAs Total (mM)	pH
Control	0	0.3 ± 0.5	2.0 ± 0.8	1.6 ± 0.7	0.4 ± 0.4	2.34 ± 0.14	4.7 ± 1.1	6.9 ± 0.04
	4	0.04 ± 0.08	2.5 ± 0.6	0.5 ± 0.9	0.3 ± 0.2	2.5 ± 0.4	5.4 ± 1.3	6.9 ± 0.05
	7	0.0 ± 0.0	5.8 ± 2.5	0.2± 0.3	1.0 ± 0.7	3.5 ± 0.4	10.3 ± 3.5	7.1 ± 0.07
	10	0.0 ± 0.0	8.5 ± 3.3	0.0 ± 0.00	1.6 ± 1.0	4.3 ± 0.4	14.8 ± 3.9	7.2 ± 0.2
	24	0.0 ± 0.0	18.8 ± 1.2	0.0 ± 0.0	5.3 ± 0.6	6.6 ± 0.3	30.8 ± 1.3	7.1 ± 0.7
	30	0.0 ± 0.0	17.3 ± 1.8	0.17 ± 0.3	4.7 ± 0.4	5.5 ± 1.1	27.6 ± 3.2	6.7 ± 0.08
FOS	0	0.44 ± 0.07 ^a^	3.4 ± 2.2 ^a^	1.8 ± 0.3 ^a^	1.3 ± 1.5 ^a^	2.5 ± 0.4 ^a^	7.2 ± 3.7 ^a^	6.9 ± 0.04
	4	0.8 ± 0.4 ^a^	4.4 ± 2.0 ^a^	2.6 ± 2.1 ^a^	2.5 ± 2.7 ^a^	2.8 ± 0.7 ^a^	9.7 ± 0.75 ^a^	6.7 ± 0.1
	7	8.3 ± 6.5 ^a^	23.2 ± 12.0 ^a^	10.2 ± 5.2 ^a^	6.2 ± 4.1 ^a^	5.6 ± 2,7 ^a^	35.0 ± 17.0 ^a^	6.1 ± 1.0
	10	6.7 ± 7.3 ^a^	33.5 ± 5.5 ^a^	12.5 ± 4.6 ^a.b^	11.6 ± 3.1 ^a^	9.4 ± 2,4 ^a^	54.4 ± 7.7 ^a^	5.1 ± 0.8
	24	5.6 ± 9.4 ^a^	40.4 ± 3.0 ^a^	14.6 ± 2.3 ^a^	18.4 ± 3.3 ^a^	13.7 ± 5.6 ^ab^	72.5 ± 6.8 ^a^	5.0 ± 0.3
	30	4.4 ± 7.6 ^a^	38.3 ± 4.5 ^a^	9.5 ± 2.4 ^a^	15.0 ± 5.4 ^a.b^	9.7 ± 4.9 ^ab^	63.0 ± 13.3 ^a^	4.9 ± 0.2
F2	0	0.9 ± 1.5	3.6 ± 2.1 ^a^	4.0 ± 1.7 ^a^	0.17 ± 0.3 ^a^	2.1 ± 0.2 ^a^	5.9 ± 2.2 ^a^	7.4 ± 0.03
	4	1.0 ± 0.29	6.1 ± 2.3 ^a^	2.1 ± 0.9 ^a^	0.6 ± 0.4 ^a^	2.9 ± 0.8 ^a^	9.6 ± 3.2 ^a^	7.2 ± 0.03
	7	3.1 ± 1.60	29.7 ± 12.9 ^a.b^	1.9 ± 0.95 ^b^	3.8 ± 2.6 ^a^	6.6 ± 2.6 ^a^	40.2 ± 17.3 ^a^	6.5 ± 0.2
	10	2.5 ± 2.6	51.5 ± 1.5 ^b^	4.1 ± 1.2 ^c^	8.2 ± 3.5 ^a^	10.6 ± 1.3 ^a^	70.3 ± 4.2 ^a^	5.8 ± 0.1
	24	0.0 ± 0.0	63.1 ± 3.8 ^c^	0.6 ± 0.5 ^c^	12.5 ± 2.3 ^b^	11.9 ± 3.0 ^a,b^	87.5 ± 3.2 ^ac^	6.0 ± 0.08
	30	0.0 ± 0.0	65.2 ± 5.2 ^b.c^	0 ± 0 ^b^	12.7 ± 3.2 ^a.b^	12.2 ± 2.5 ^a,b^	90.1 ± 2.3 ^b^	5.9 ± 0.1

Data are presented as mean ± standard deviation of three different donors shown in parentheses. Different letters indicate significant differences (*p* < 0.05) for the same acid and time.

## Data Availability

Data is contained within the article and supplementary material.

## Supplementary Materials

The following supporting information can be downloaded at https://www.mdpi.com/article/10.3390/foods12010083/s1, Figure S1. Spectrum MALDI-TOF-MS for F2 compounds.
