# Supplementary material for: In Vitro Assessment of the Prebiotic Potential of Xylooligosaccharides from Barley Straw
Source: Foods. 2022 Dec 23;12(1):83. doi: 10.3390/foods12010083 (PMC9818743; doi:10.3390/foods12010083)
Supplement: Supplementary file 1 [file foods-12-00083-s001.zip › foods-1999887-supplementary.pdf]

Supporting Information

In vitro assessment of the prebiotic potential of xylooligosaccharides from barley straw

Cristina Álvarez[a*], Alberto González[a], Ignacio Ballesteros[a] , Beatriz Gullón[b], María José Negro[a]

[a]Advanced Biofuels and Bioproducts Unit, Energy Department-CIEMAT, 28040-Madrid, Spain

[b]Department of Chemical Engineering, Faculty of Science, University of Vigo (Campus Ourense), As Lagoas, 32004 Ourense, Spain

Corresponding author: cristina.alvarez@ciemat.es

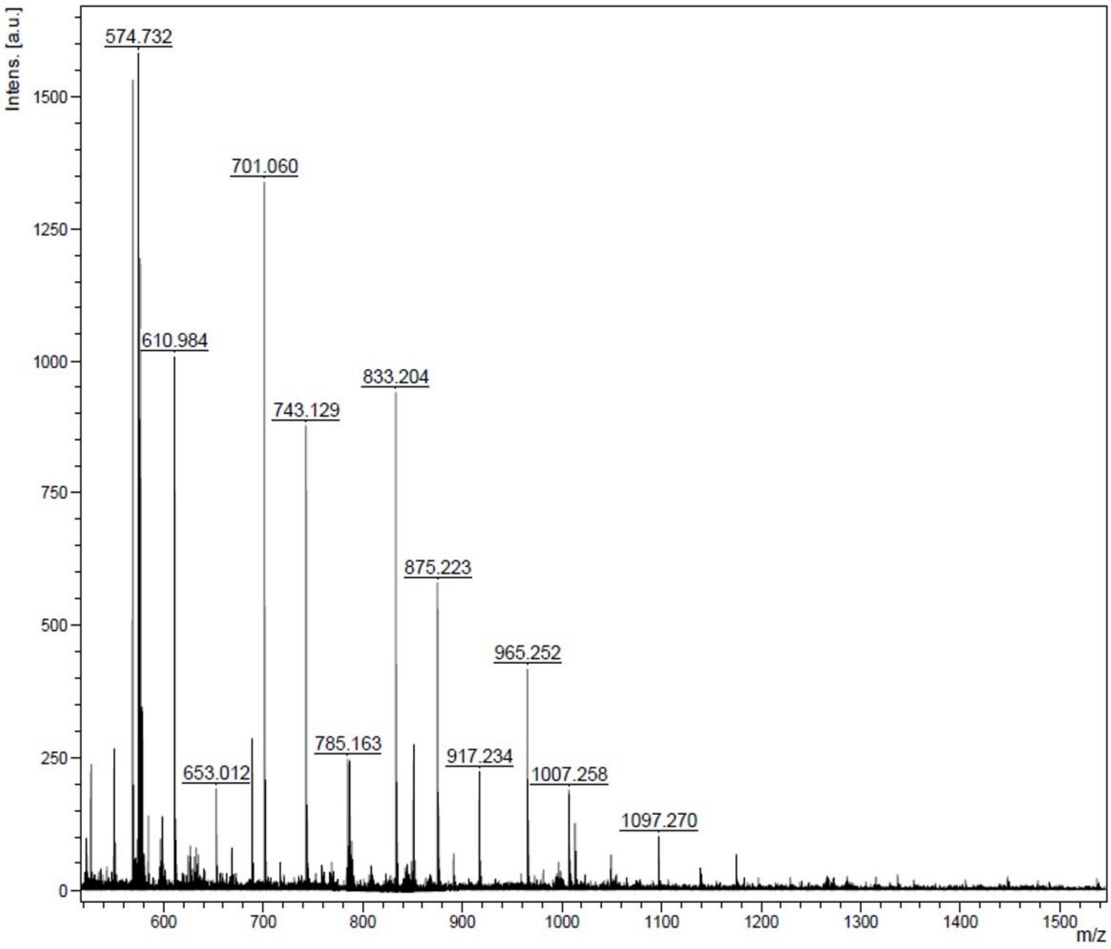

Figure S1: Spectrum MALDI-TOF-MS for F2 compounds
